# The National Institute for Health Research Hyperacute Stroke Research Centres and the ENCHANTED trial: the impact of enhanced research infrastructure on trial metrics and patient outcomes

**DOI:** 10.1186/s12961-019-0417-2

**Published:** 2019-02-13

**Authors:** Thompson G. Robinson, Xia Wang, Alice C. Durham, Gary A. Ford, Joy Liao, Sine Littlewood, Christine Roffe, Philip White, John Chalmers, Craig S. Anderson, on behalf of the ENCHANTED Investigators

**Affiliations:** 1 Department of Cardiovascular Sciences, University of Leicester, Leicester, United Kingdom; 2 NIHR Leicester Biomedical Research Centre, Leicester, United Kingdom; 3 The George Institute for Global Health, Faculty of Medicine, University of New South Wales, Sydney, NSW Australia; 4 Oxford University Hospitals NHS Foundation Trust and Radcliffe Department of Medicine, University of Oxford, Oxford, United Kingdom; 5 NIHR Specialty Cluster A Co-ordinating Centre, Imperial College London, London, United Kingdom; 6 NIHR Clinical Research Network National Co-ordinating Centre, Leeds, United Kingdom; 7 Stroke Research in Stoke Institute for Applied Clinical Studies, Keele University, Staffordshire, United Kingdom; 8 Institute of Neuroscience Newcastle University and Newcastle upon Tyne Hospitals NHS Trust, Newcastle upon Tyne, United Kingdom; 9 Neurology Department, Royal Prince Alfred Hospital, Sydney, NSW Australia; 10 The George Institute China at Peking University Health Sciences Center, Beijing, China; 11 BHF Cardiovascular Research Centre, Glenfield Hospital, Groby Road, Leicester, LE3 9QP United Kingdom

**Keywords:** Acute ischaemic stroke, alteplase, clinical trials, symptomatic intracerebral haemorrhage, thrombolysis

## Abstract

**Background:**

The English National Institute for Health Research Clinical Research Network first established Hyperacute Stroke Research Centres (HSRCs) in 2010 to support multicentre hyperacute (< 9 h) and complex stroke research. We assessed the impact of this investment on research performance and patient outcomes in a post-hoc analysis of country-specific data from a large multicentre clinical trial.

**Methods:**

Comparisons of baseline, outcome and trial metric data were made for participants recruited to the alteplase-dose arm of the international Enhanced Control of Hypertension and Thrombolysis Stroke study (ENCHANTED) at National Institute for Health Research Clinical Research Network HSRCs and non-HSRCs between June 2012 and October 2015.

**Results:**

Among 774 ENCHANTED United Kingdom participants (41% female; mean age 72 years), 502 (64.9%) were recruited from nine HSRCs and 272 (35.1%) from 24 non-HSRCs. HSRCs had higher monthly recruitment rates (median 1.5, interquartile interval 1.4–2.2 vs. 0.7, 0.5–1.3; *p* = 0.01) and shorter randomisation-to-treatment times (2.6 vs. 3.1 min; *p* = 0.01) compared to non-HSRCs. HSRC participants were younger and had milder stroke severity, but clinically important between-group differences in 90-day death or disability outcomes remained after adjustment for minimisation criteria and important baseline variables at randomisation, whether defined by ordinal modified Rankin scale score shift (adjusted OR 0.82, 95% CI 0.62–1.08; *p* = 0.15), scores 2 to 6 (adjusted OR 0.71, 95% CI 0.50–1.01; *p* = 0.05), or scores 3 to 6 (adjusted OR 0.82, 95% CI 0.57–1.17; *p* = 0.27). There was no significant difference in symptomatic intracerebral haemorrhage, nor heterogeneity in the comparative treatment effects between low- and standard-dose alteplase by HSRCs or non-HSRCs.

**Conclusions:**

Infrastructure investment in HSRCs was associated with improved research performance metrics, particularly recruitment and time to treatment with clinically important, though not statistically significant, improvements in patient outcomes.

**Trial Registration:**

Unique identifier: NCT01422616.

**Electronic supplementary material:**

The online version of this article (10.1186/s12961-019-0417-2) contains supplementary material, which is available to authorized users.

## Background

Intravenous (iv) alteplase (recombinant tissue plasminogen activator) is the approved medical reperfusion treatment for patients with acute ischaemic stroke and the earlier the treatment is given, the greater the benefit [1]. However, concerns over risks of intracranial haemorrhage have led to lower doses of alteplase being used in many Asian countries [2] after a lower dose (0.6 mg/kg) was approved in Japan. The Enhanced Control of Hypertension and Thrombolysis Stroke Study (ENCHANTED) assessed a low dose (0.6 mg/kg body weight) compared to the standard dose (0.9 mg/kg) of iv alteplase in acute ischaemic stroke patients fulfilling standard criteria for thrombolysis (‘clot-busting’ therapy) in improving treatment efficacy through reduced 90-day death and disability, and safety through reduced symptomatic intracerebral haemorrhage (bleeding associated with neurological deterioration or death) [3]. The primary outcome of the study was to demonstrate non-inferiority of low- compared to standard-dose alteplase on 90-day death and disability, defined by scores 2 to 6 on the modified Rankin scale (mRS). The mRS is a global seven-level assessment of disability, where scores of 0 to 1 indicate a favourable outcome with/without symptoms but no disability; 2 to 5 indicate increasing levels of disability and dependency, requiring no (2), weekly (3), daily (4), and 24-h (5) physical help; and 6 indicates death. Whilst low-dose alteplase was not non-inferior to the standard dose, it was clearly non-inferior on a secondary efficacy outcome of shift (‘improvement’) in measures of daily function according to the full range of scores on the mRS scores, and reduced the risk of symptomatic intracerebral haemorrhage [3].

The United Kingdom had the second highest patient recruitment (*n* = 774, 23.3%) to the ENCHANTED alteplase dose-arm after China, supported by the National Institute for Health Research (NIHR) Clinical Research Network infrastructure for clinical research through the National Health Service (NHS). In particular, NIHR has made significant investment in eight (expanding to 10 during the conduct of ENCHANTED) Hyperacute Stroke Research Centres (HSRCs), established in 2010 with an initial investment of £3.66 million over 3 years. These high patient volume centres (> 1000 stroke admissions per annum) provide multi-disciplinary expertise in hyperacute stroke clinical management (including engagement of pre-hospital, emergency department, interventional neuroradiology, neuroradiology, neuro-critical care, and neurosurgical staff), 24/7 availability of advanced neuroimaging, 7-day per week extended hours (07:00 to 22:00, minimum) resident research staffing, and a track record of performing against specialty performance objectives (in particular recruitment to the hyperacute (< 9 h of stroke onset) and complex trial portfolio (a trial requiring complex diagnostics, or surgery/complex intervention, or phase 1 or 2 commercial study)). In addition, the HSRC selection process includes a review of recent scores on the Sentinel Stroke National Audit Programme, particularly related to hyperacute process metrics, including door-to-scan time, rates of admission to Hyperacute Stroke Units within 4 h and thrombolysis rates, where scores at the highest level (A or B) should be consistently achieved. HSRCs are subject to annual review by an independent panel to maintain their accreditation. The return on such investment in infrastructure might extend beyond improved research performance; therefore, whilst we primarily evaluated the impact of HSRCs on trial metrics, we also assessed the effect on patient outcomes in NIHR Clinical Research Network sites participating in ENCHANTED in England.

## Methods

### Design

The ENCHANTED trial was an international, multi-centre, prospective, randomised, open-label, blinded-endpoint trial with a 2 × 2 partial-factorial design to assess the effectiveness of low- versus standard-dose alteplase (completed arm) and more intensive versus guideline-recommended control of blood pressure (ongoing arm), the details of which are outlined in detail elsewhere [3, 4]. In brief, patients admitted to hospital with a clinical diagnosis of acute ischaemic stroke confirmed on brain imaging and fulfilling local criteria for thrombolysis treatment administered within 4.5 h of symptom onset, were randomly assigned to the respective dose arm between 18 June 2012 and 14 October 2015. Randomised patients received low-dose (0.6 mg/kg; 15% as bolus, 85% as infusion over 1 h; maximum dose 60 mg) or standard-dose (0.9 mg/kg; 10% as bolus, 90% as infusion over 1 h; maximum dose 90 mg) iv alteplase. Otherwise, all patients received active care and best practice management according to local guidelines. The study protocol was approved by the appropriate ethics committee at each participating centre, and written informed consent was obtained from the patient or an appropriate surrogate.

### Procedures

Key demographic and clinical characteristics were recorded at the time of enrolment. Stroke severity was measured using the National Institutes of Health Stroke Scale at baseline, at 24 h, and on day 7 (or earlier, upon discharge from hospital). Uncompressed digital images of all baseline and follow-up brain scans (CT, MRI and angiogram) were collected, with details of transfer, interpretation and intracranial haemorrhage definitions provided (Additional file 1).

As previously stated, the primary clinical outcome was the combined endpoint of death or disability at 90 days, defined by scores of 2 to 6 on the mRS. Other efficacy outcomes included an ordinal mRS shift and the combined endpoint of death or major disability (mRS scores of 3 to 6) at 90 days. The secondary (safety) outcome was symptomatic intracerebral haemorrhage, defined according to several criteria from other studies (Additional file 1). Additional new analyses in this manuscript focus on trial performance metrics, including total recruitment, monthly recruitment rate, and times to randomisation and treatment.

### Statistical analysis

The association of HSRC recruitment on global functional outcome (analysis of the full range of day 90 mRS scores) was estimated using ordinal logistic regression after the assumption of proportionality of the odds was confirmed from a likelihood ratio test. Adjustment was made for the ENCHANTED pre-specified minimisation variables and several baseline covariates at randomisation, and additionally for aspects of management over the first 7 days after hospital admission. In patients from HSRCs and non-HSRCs, the heterogeneity of the alteplase treatment effect was tested by adding an interaction term to the statistical models. Data are reported with odds ratios (ORs) and 95% confidence intervals (CIs). A two-sided *p* value of less than 0.05 was considered statistically significant. SAS version 9.3 (SAS Institute, Cary, NC) was used for all analyses.

### Role of the funding source

The sponsors had no role in the study design, data collection, data analysis, data interpretation or writing of the report. All authors had full access to the study data. The corresponding author had final responsibility for the decision to submit the paper for publication.

## Results

These analyses included 774 patients (41% female; mean age 72 years) randomised to the ENCHANTED trial in the United Kingdom (England 768, non-England 6), including 9 of the 10 established HSRCs (one site did not participate in ENCHANTED). Although HSRCs comprised only 27% (9/33) of recruiting sites, they contributed nearly two-thirds (502, 64.9%) of participants and had a significantly higher median monthly recruitment rate than non-HSRCs (1.5 (interquartile interval 1.4–2.2) vs. 0.7 (0.5–1.3)). With respect to performance on other trial metrics, Table 1 shows that HSRC patients had significantly shorter randomisation-to-treatment times, as well as differences in other aspects of management, including angiography and endovascular therapy. Table 2 shows that participants recruited from HSRCs were significantly younger, and had milder severity of stroke, as assessed by National Institutes of Health Stroke Scale or Glasgow coma score. HSRC patients were also significantly less likely to develop fever, receive antiplatelet therapy in the first 24 h after thrombolysis, or have rehabilitation in the first 7 days, but were more likely to receive subcutaneous heparin (Additional file 2: Table S1).

**Table 1:** Selected trial metrics and management of patients by type of stroke research centre

	All United Kingdom (*n* = 774)	HSRC (*n* = 502)	Non-HSRC (*n* = 272)	*p* value
Number of recruiting centres (*n*)	33	9	24	
Monthly recruitment rate (median, IQR)	1.0 (0.6–1.5)	1.5 (1.4–2.2)	0.7 (0.5–1.3)	0.01
Time from stroke onset to treatment, mins (median, IQR)	139 (110–175)	135.5 (110–178)	140.0 (111–174)	0.65
Time from randomisation to treatment, mins (median, IQR)	2.8 (0.2–5.4)	2.6 (−0.6 to 5.3)	3.1 (1.1–5.7)	0.01
Alteplase given (*n*/*N*, %)	765/774 (98.8)	498/502 (99.2)	267/272 (98.2)	0.20
Cerebral angiogram (*n*/*N*, %)	22/774 (2.8)	20/502 (4.0)	2/272 (0.7)	0.009
Occluded vessel (*n*/*N*, %)	18/22 (81.8)	17/20 (85.0)	1/2 (50.0)	0.22
Endovascular therapy (*n*/*N*, %)	16/22 (72.7)	15/20 (75.0)	1/2 (50.0)	0.45

*HSRC* hyperacute stroke research centre, *IQR* interquartile range

**Table 2:** Baseline characteristics of patients by type of stroke research centre

	All United Kingdom (*n* = 774)	HSRC (*n* = 502)	Non-HSRC (*n* = 272)	*p* value
Time from stroke onset to randomisation, hours (median, IQR)	2.3 (1.8–2.9)	2.2 (1.8–2.9)	2.3 (1.8–2.8)	0.82
Age, years (mean, SD)	72 (14)	71 (14)	75 (13)	0.0002
Female (*n*/*N*, %)	318/774 (41.1)	203/502 (40.4)	115/272 (42.3)	0.62
Non-Asian ethnicity (*n*/*N*, %)	757/774 (97.8)	490/502 (97.6)	267/272 (98.2)	0.62
Systolic BP, mmHg (mean, SD)	151 (20)	151 (19)	152 (20)	0.52
Heart rate, bpm (mean, SD)	80 (18)	82 (18)	77 (17)	0.0003
NIHSS score (median, IQR)	7 (5–13)	7 (5–11)	8 (5–14)	0.01
GCS score (median, IQR)	15 (14–15)	15 (14–15)	15 (14–15)	0.01
History of hypertension (*n*/*N*, %)	472/774 (61.0)	299/502 (59.6)	173/272 (63.6)	0.27
History of coronary artery disease (*n*/*N*, %)	112/774 (14.5)	73/502 (14.5)	39/272 (14.3)	0.94
AF confirmed on ECG (*n*/*N*, %)	188/772 (24.4)	117/500 (23.4)	71/272 (26.1)	0.40
Hypercholesterolaemia (*n*/*N*, %)	260/774 (33.6)	161/502 (32.1)	99/272 (36.4)	0.22
Current smoker (*n*/*N*, %)	124/770 (16.1)	92/501 (18.4)	32/269 (11.9)	0.02
Pre-stroke symptoms on the mRS (*n*/*N*, %)	240/773 (31.0)	149/501 (29.7)	91/272 (33.5)	0.29
Antihypertensive therapy (*n*/*N*, %)	429/774 (55.4)	264/502 (52.6)	165/272 (60.7)	0.03
Warfarin anticoagulation (*n*/*N*, %)	23/773 (3.0)	18/501 (3.6)	5/272 (1.8)	0.17
Aspirin/other antiplatelet (*n*/*N*, %)	273/773 (35.3)	178/501 (35.5)	95/272 (34.9)	0.87
Statin/other lipid lowering (*n*/*N*, %)	103/773 (13.3)	66/501 (13.2)	37/272 (13.6)	0.87
Brain imaging features				
CT scan used (*n*/*N*, %)	769/774 (99.4)	502/502 (100)	267/272 (98.2)	0.002
MRI scan used (*n*/*N*, %)	8/774 (1.0)	4/502 (0.8)	4/272 (1.5)	0.38
Visible early ischaemic changes (*n*/*N*, %)	230/774 (29.7)	156/502 (31.1)	74/272 (15.1)	0.26
Visible cerebral infarction (*n*/*N*, %)	144/774 (18.6)	103/502 (20.5)	1/272 (0.4)	0.06
Final diagnosis				
Non-stroke (*n*/*N*, %)	50/768 (6.5)	33/498 (6.6)	17/270 (6.3)	0.86
Large artery occlusion (*n*/*N*, %)	170/713 (23.8)	129/461 (28.0)	41/252 (16.3)	0.003
Small vessel disease (*n*/*N*, %)	139/713 (19.5)	91/461 (19.7)	48/252 (19.0)	
Cardioembolism (*n*/*N*, %)	181/713 (25.4)	106/461 (23.0)	75/252 (29.8)	
Other or uncertain aetiology (*n*/*N*, %)	223/713 (31.3)	135/461 (29.3)	88/252 (34.9)	

*HSRC* Hyperacute Stroke Research Centre, *BP* blood pressure, *NIHSS* National Institutes of Health Stroke Scale, *GCS* Glasgow coma scale, *AF* atrial fibrillation, *mRS* modified Rankin scale

Compared to non-HSRC patients, HSRC-treated patients were significantly less likely to be dead or disabled at 90 days, whether defined by ordinal shift (unadjusted OR 0.68, 95% CI 0.52–0.89; *p* = 0.005), scores 2 to 6 (unadjusted OR 0.63, 95% CI 0.46–0.86; *p* < 0.0001), or scores 3 to 6 (unadjusted OR 0.69, 95% CI 0.50–0.82; *p* = 0.017) on the mRS. There remained clinically important, though not statistically significant differences in mortality or disability outcomes at 90 days after adjustment for the minimisation criteria and baseline prognostic variables, whether defined by ordinal shift (adjusted OR 0.82, 95% CI 0.62–1.08; *p* = 0.15), scores 2 to 6 (adjusted OR 0.71, 95% CI 0.50–1.01; *p* = 0.05), or scores 3 to 6 (adjusted OR 0.82, 95% CI 0.57–1.17; *p* = 0.27). There were no significant differences after further adjustment for imbalances in early management (Table 3).

**Table 3:** Clinical outcomes at 90 days in patients by type of stroke research centre

	*n* (%)	Unadjusted OR (95% CI)	*p* value	Adjusted OR^a^ (95% CI)	*p* value	Adjusted OR^b^ (95% CI)	*p* trend
Death or disability (mRS 2 to 6)							
Non-HSRC	165/256 (64.5)	1.0	0.004	1.0	0.05	1.0	0.87
HSRC	249/467 (53.3)	0.63 (0.46–0.86)		0.71 (0.50–1.01)		0.97 (0.64–1.46)	
Death or disability (mRS 3 to 6)							
Non-HSRC	121/256 (47.3)	1.0	0.017	1.0	0.27	1.0	0.82
HSRC	178/467 (38.1)	0.69 (0.50–0.82)		0.82 (0.57–1.17)		1.05 (0.68–1.62)	
Death							
Non-HSRC	34/272 (12.5)	1.0	0.24	1.0	0.60	1.0	0.58
HSRC	49/502 (9.8)	0.76 (0.48–1.21)		0.87 (0.51–1.47)		1.21 (0.61–2.39)	
mRS categories							
Non-HSRC		1.0	0.005	1.0	0.15	1.0	0.77
0	37 (14.5)						
1	54 (21.1)						
2	44 (17.2)						
3	45 (17.6)						
4	23 (9.0)						
5	19 (7.4)						
6	34 (13.3)						
HSRC		0.68 (0.52–0.89)		0.82 (0.62–1.08)		1.05 (0.77–1.43)	
0	100 (21.4)						
1	118 (25.3)						
2	71 (15.2)						
3	61 (13.1)						
4	42 (9.0)						
5	26 (5.6)						
6	34 (13.3)						

*CI* confidence interval, *HSRC* hyperacute stroke research centre, *mRS* modified Rankin score, *OR* odds ratio, *aOR* adjusted odds ratio

^a^Model 1: adjusted analysis for minimisation variables including National Institutes of Health Stroke Scale score and time from stroke onset to randomisation, and baseline variables: age, sex, ethnicity, systolic blood pressure, heart rate, hypercholesterolaemia, current smoker, premorbid mRS, premorbid use of antihypertensive therapy, aspirin or other antiplatelet agent, and randomised treatment (low dose versus standard dose)

^b^Model 2: as Model 1, plus systolic blood pressure at 24 h, fever occurrence, nasogastric feeding given, subcutaneous heparin used, patient mobilised by therapist, any stroke unit admission, any neurosurgery performed, and any rehabilitation given

Moreover, no significant differences were seen in symptomatic intracerebral haemorrhage between HSRC and non-HSRC patient groups across a broad range of definitions (Additional file 2: Table S2), nor were there any significant differences in the treatment effect (Additional file 2: Tables S3 and S4) or safety (Additional file 2: Table S5) between low- and standard-dose alteplase between HSRC and non-HSRC treated patients.

## Discussion

In these country-specific secondary analyses of the ENCHANTED trial of low- versus standard-dose alteplase in thrombolysis-eligible acute ischaemic stroke patients, we have shown significantly higher recruitment rates and improved trial performance metrics in relation to local research infrastructure investment. HSRCs were established with additional funding to support hyperacute complex stroke trials, and while only 9 of the 33 United Kingdom recruiting centres (England 31, non-England 2) in ENCHANTED were HSRC designated, they recruited nearly two-thirds of all the patients in the United Kingdom, and with faster recruitment and shorter randomisation-to-treatment times. Furthermore, whilst 90% of English HSRCs participated in ENCHANTED, the participation rate was far lower amongst the English non-HSRC sites, at 21% (24/117). HSRC-treated patients had significantly better clinical outcomes in the unadjusted analyses, which remained clinically, though not quite statistically, significant after adjustment for minimisation criteria and differences in baseline characteristics. No differences were seen compared to non-HSRC-treated patients after adjustment for early management, which may reflect better care processes in HSRCs.

The importance of research is explicitly stated in The Handbook to the NHS Constitution in the United Kingdom [5]. Indeed, the NIHR Clinical Research Network was specifically established with the aim of improving the health and well-being of the nation, through research focused on the needs of patients and the public. In the field of stroke, additional funding in the HSRC network was directed to deliver hyperacute (randomisation < 9 h of symptom onset) and/or complex studies (using advanced neuroimaging or other diagnostics, interventional neuroradiology or neurosurgery). An assessment of any positive impact of this research infrastructure investment on research performance measures and health outcomes would be important to demonstrate to NHS commissioners and providers the value of research infrastructure funding. To our knowledge, this is the first such evaluation on the return on this HSRC investment.

Analyses of practice-based research networks in the United States have shown improved clinical outcomes in participating practices [6], whilst previous NIHR data analyses have shown that Trusts with the lowest mortality for adult non-elective admissions had higher levels of research patient recruitment and funding than those with expected or higher mortality rates [7]. The survival benefits for research participants and other patients separately persist after adjustment for staffing and other hospital structural factors such as medical and staffing ratios per bed, critical care versus general bed ratios, and affiliations with a university [7]. Our study provides further support for positive impact of research investment on research participation, trial performance metrics and patient recruitment.

In addition, though we were unable to clearly demonstrate a statistically significant association between research investment and improved patient outcomes in adjusted (as opposed to unadjusted) analyses, this is most likely related to the sample size and thus the limited statistical power to confirm modest but still clinically important differences. Indeed, even after adjustment for minimisation criteria and baseline differences, we were able to demonstrate a borderline statistical, but clinically significant, 29% reduction in 90-day death and disability, defined by a mRS of 2 to 6. Jonker and Fisher [8] were also unable to show that the degree of NIHR portfolio clinical research activity was significantly related to risk rating for overall performance by the Care Quality Commission. Nonetheless, another study of high-risk conditions, including stroke, has shown a significant correlation between academic output, as measured by citations per hospital admission, and overall mortality [9]. Furthermore, a study of colorectal cancer outcome by the NIHR Cancer Research Network reported a significant reduction in post-operative mortality and improved 5-year survival in research-active centres [10]. Indeed, there are other examples of improved patient outcome and adherence to guidelines in hospitals participating in related clinical trials compared to other hospitals, for example, in relation to the management of non-ST-segment elevation acute coronary syndromes [11].

Several possible mechanisms have been proposed for why clinical research may improve health outcomes, including enhanced infrastructure and organisation, multidisciplinary and institutional collaboration, education and training, specialisation, and care processes, particularly in the uptake and adherence to guideline-recommended care [12, 13]. In particular, HSRCs provide enhanced infrastructure, including 7-day per week extended hours (07:00 to 22:00, minimum) resident research staffing and improved collaboration between key specialties in hyperacute stroke management. These mechanisms are supported by two recent systematic reviews, which concluded that a strong research culture has patient, staff and organisational benefits [14], and that institution and clinician engagement in clinical trials has greater adherence to guidelines and better outcomes [15]. Whilst we were unable to explore these themes retrospectively, a qualitative study has reinforced their relevance in an NIHR context at the NIHR Oxford Biomedical Research Centre [16]. Another potential explanation for the research and clinical benefits of HSRCs on patient care processes and outcomes may relate to a ‘volume effect’ [17]. One of the criteria included in HSRC designation is the number of annual stroke admissions, with the average annual admission rate in HSRCs being higher compared to non-HSRCs (1713 (range 820–2920) vs. 971 (288–1556)) [18].

We acknowledge that our study is limited by being based on data derived from a single country where the open-label trial design may have introduced various biases despite our efforts at concealment of treatment allocation, assessment of adverse events and blinded evaluation of clinical outcomes using established criteria. Moreover, as the ENCHANTED trial included patients with generally milder stroke severity with a slightly longer treatment delay from onset than in previous trials or registries [19], there may be further concerns over the generalisability of these data, and imprecision in the estimates of the effects may have arisen from variability in the assessment of the mRS [20]. In addition, the proportion of female patients is lower than might be expected given the age demographic of stroke, and highlights regional differences in sex distribution between registry and trial populations, with male predominance being previously reported in stroke thrombolysis trials [21]. Finally, it is difficult to determine if the improved research performance of HSRCs was related to the research infrastructure investment associated with their designation as an HSRC, or whether these were already high performing research centres or clinical Hyperacute Stroke Units. Only one centre changed status from a non-HSRC to a HSRC during the trial, with average monthly recruitment rates of 1.3 and 2.4 over 29 months before and 4.5 months after change in HSRC status, respectively. However, five HSRCs participated in the Third International Stroke Trial, between May 2000 and July 2011 [22], which is a similar trial comparing iv thrombolysis with control treatment up to 6 h following acute ischaemic stroke onset. Average monthly recruitment rates increased by approximately 50% from 0.8 and 1.1 between April 2007 and March 2010 (pre-HSRC status) and April 2010 to July 2011 (HSRC status), respectively.

## Conclusions

We have demonstrated a positive impact of research infrastructure investment, with significant improvements in overall recruitment and other trial performance metrics in HSRCs, including monthly recruitment rates and time to randomisation and treatment. This also led to broader clinically important benefits in patient outcomes, even after adjustment for differences in minimisation criteria and baseline characteristics, though these did not reach statistical significance. This should provide confidence to commissioners and providers of the importance of research investment and participation, as highlighted in the NHS Constitution.

## Additional files

Additional file 1: Imaging transfer and analysis, and definitions of symptomatic intracerebral haemorrhage. (DOCX 40 kb)

Additional file 2: **Table S1.** Use of alteplase and management details from randomisation to day 7 by hyperacute stroke research centres (HSRCs) and non-HSRCs. **Table S2.** Key secondary outcome of symptomatic intracerebral haemorrhage across all definitions by HSRCs and non-HSRCs. **Table S3.** Key efficacy outcomes by randomised treatment and HSRCs and non-HSRCs. **Table S4.** Key efficacy outcomes by randomised treatment and HSRCs and non-HSRCs. **Table S5.** Key safety outcome of symptomatic intracerebral haemorrhage by randomised treatment and HSRCs and non-HSRCs. (DOCX 49 kb)

## Abbreviations

CI: confidence interval
ENCHANTED: Enhanced Control of Hypertension and Thrombolysis Stroke Study
HSRC: Hyperacute Stroke Research Centre
iv: intravenous
mRS: modified Rankin scale
NHS: National Health Service
NIHR: National Institute for Health Research
OR: odds ratio
