# Supplementary material for: The National Institute for Health Research Hyperacute Stroke Research Centres and the ENCHANTED trial: the impact of enhanced research infrastructure on trial metrics and patient outcomes
Source: Health Res Policy Syst. 2019 Feb 13;17:19. doi: 10.1186/s12961-019-0417-2 (PMC6375185; doi:10.1186/s12961-019-0417-2)
Supplement: Supplementary file 1 — Imaging transfer and analysis, and definitions of symptomatic intracerebral haemorrhage. (DOCX 40 kb) [file 12961_2019_417_MOESM1_ESM.docx]

**The National Institute for Health Research Hyperacute Stroke Research Centres and the ENCHANTED trial: the impact of enhanced research infrastructure on trial metrics and patient outcomes**

**Robinson TG et al**

**Additional file 1**

**Imaging transfer and analysis, and definitions of symptomatic intracerebral haemorrhage**

Uncompressed digital images of all baseline and follow-up digital CT, MRI and angiogram images, were collected in DICOM format on a CD-ROM identified only with the patient’s unique study number, and analysed centrally for any intracranial haemorrhage by independent assessors blinded to clinical data, treatment, and date and sequence of scan. Assessors graded any identified haemorrhage as intracerebral using a range of standard definitions (see below) and subarachnoid, intraventricular, subdural or other.

For intracererbal haemorrhage, bleeding was coded as HI1 (small petechiae along infarct margins), HI2 (confluent petechiae within infarcted area without space-occupying effect), PH1 (blood clot(s) in <30% of infarcted area with slight space-occupying effect) and PH2 (blood clot(s) in >30% of infarcted area with substantial space-occupying effect). In addition, independent assessors were asked to adjudicate if haemorrhage was the predominant cause of neurological worsening, and if there was evidence of midline shift. These assessments enabled the following definitions of symptomatic intracerebral haemorrhage (sICH) to be adjudicated: Safe Implementation of Thrombolysis in Stroke Monitoring Study (SITS-MOST): large or remote parenchymal ICH (type 2, defined as greater than 30% of the infarcted area affected by haemorrhage with mass effect or extension outside the infarct) combined with neurological deterioration (>4 points on the NIHSS) or leading to death within 24 to 36 hours [Wahlgren et al, 2007]; any ICH associated with neurological deterioration (>1 point change in NIHSS score) from baseline or death within 24 to 36 hours (NINDS) [NINDS Study Group, 1995]; any ICH with neurological deterioration (>4 points on the NIHSS) from baseline or death within 24 to 36 hours (ECASS2) [Hacke et al, 1998]; any ICH with neurological deterioration (>4 points increase on the NIHSS) from baseline or death within 36 hours (ECASS3) [Hacke et al, 2008]; either significant ICH (local or distant from the infarct) or significant haemorrhagic transformation of an infarct on brain imaging with clinically significant deterioration or death within the first 7 days of treatment (IST3) [IST-3 Collaborative Group, 2012]; and fatal ICH, any type 2 parenchymal ICH and death within 7 days.

**References**

Wahlgren N, Ahmed N, Davalos S, et al. Thrombolysis with alteplase for acute ischaemic stroke in the Safe Implementation of Thrombolysis in Stroke-Monitoring Study (SITS-MOST): an observational study Lancet 2007; 369: 275-282.

The National Institute of Neurological Disorders and Stroke rt-PA Stroke Study Group. Tissue plasminogen activator for acute ischemic stroke. N Engl J Med 1995; 333:1581-1587.

Hacke W, Kaste M, Fieschi C, et al. Randomised double-blind placebo controlled trial of thrombolytic therapy with intravenous alteplase in acute ischemic stroke (ECASS II). Lancet 1998; 352: 1245-1251.

Hacke W, Kaste M, Bluhmki E, et al. Thrombolysis with alteplase 3 to 4.5 hours after acute ischemic stroke. N Engl J Med 2008; 359: 1317-1329.

The IST-3 Collaborative Group. The benefits and harms of intravenous thrombolysis with recombinant tissue plasminogen activator within 6 h of acute ischaemic stroke (the third international stroke trial [IST-3]): a randomized controlled trial. Lancet 2012; 379:2352-2363.
