# Supplementary material for: The National Institute for Health Research Hyperacute Stroke Research Centres and the ENCHANTED trial: the impact of enhanced research infrastructure on trial metrics and patient outcomes
Source: Health Res Policy Syst. 2019 Feb 13;17:19. doi: 10.1186/s12961-019-0417-2 (PMC6375185; doi:10.1186/s12961-019-0417-2)
Supplement: Supplementary file 2 — Table S1. Use of alteplase and management details from randomisation to day 7 by hyperacute stroke research centres (HSRCs) and non-HSRCs. Table S2. Key secondary outcome of symptomatic intracerebral haemorrhage across all definitions by HSRCs and non-HSRCs. Table S3. Key efficacy outcomes by randomised treatment and HSRCs and non-HSRCs. Table S4. Key efficacy outcomes by randomised treatment and HSRCs and non-HSRCs. Table S5. Key safety outcome of symptomatic intracerebral haemorrhage by randomised treatment and HSRCs and non-HSRCs. (DOCX 49 kb) [file 12961_2019_417_MOESM2_ESM.docx]

**The National Institute for Health Research Hyperacute Stroke Research Centres and the ENCHANTED trial: the impact of enhanced research infrastructure on trial metrics and patient outcomes**

**Robinson TG et al**

**Additional file 2**

Table S1 Use of alteplase and management details from randomisation to Day 7 by hyperacute stroke research centres (HSRCs) and non-HSRCs

Table S2 Key secondary outcome of symptomatic intracerebral haemorrhage across all definitions by hyperacute stroke research centres (HSRCs) and non-HSRCs

Table S3 Key efficacy outcomes by randomised treatment and hyperacute stroke research centres (HSRCs) and non-HSRCs

Table S4 Key efficacy outcomes by randomised treatment and hyperacute stroke research centres (HSRCs) and non-HSRCs

Table S5 Key safety outcome of symptomatic intracerebral haemorrhage by randomised treatment and hyperacute stroke research centres (HSRCs) and non-HSRCs

**Table S1 Use of alteplase and management details from randomisation to Day 7 by hyperacute stroke research centres (HSRCs) and non-HSRCs**

|  | All UK patients  (n=774) | HSRC patients  (n=502) | Non-HSRC patients  (n=272) | P value |
| --- | --- | --- | --- | --- |
| Thrombolysis treatment |  |  |  |  |
| Body Weight |  |  |  |  |
| Patients with estimated body weight prior to alteplase, n(%) | 774/ 774 (100%) | 502/ 502 (100%) | 272/ 272 (100%) | 0.12 |
| Estimated measurement prior to alteplase use (kg) | 77.4 (17.1) | 77.6 (16.9) | 77.0 (17.4) | 0.61 |
| Patients with direct measured body weight after alteplase use, n(%) | 665/ 774 (85.9%) | 426/ 502 (84.9%) | 239/ 272 (87.9%) | 0.25 |
| Direct measured body weight after alteplase use (kg) | 76.4 (18.1) | 77.2 (18.3) | 75.1 (17.7) | 0.16 |
| Alteplase given |  |  |  |  |
| Any given, n(%) | 765/ 774 (98.8%) | 498/ 502 (99.2%) | 267/ 272 (98.2%) | 0.20 |
| Bolus dose (mg) | 6.9 (1.3) | 6.9 (1.3) | 6.8 (1.4) | 0.47 |
| Infusion over 60 mins dose (mg) | 50.3 (15.1) | 50.4 (15.1) | 50.1 (15.1) | 0.79 |
|  |  |  |  |  |
| Management |  |  |  |  |
| Any intravenous BP lowering treatment in first 24 hours, n(%) | 111/ 772 (14.4%) | 73/ 500 (14.6%) | 38/ 272 (14.0%) | 0.81 |
| Any intravenous BP lowering treatment in days 2-7, n(%) | 55/ 768 (7.2%) | 34/ 498 (6.8%) | 21/ 270 (7.8%) | 0.63 |
| Systolic BP at 24 hours (mmHg) | 136.6 (21.0) | 135.3 (19.5) | 139.1 (23.3) | 0.03 |
| Intubation and ventilation, n(%) | 12/ 767 (1.6%) | 7/ 497 (1.4%) | 5/ 270 (1.9%) | 0.64 |
| Fever occurrence, n(%) | 116/ 767 (15.1%) | 60/ 497 (12.1%) | 56/ 270 (20.7%) | 0.001 |
| Fever treated, n(%) | 86/ 640 (13.4%) | 48/ 389 (12.3%) | 38/ 251 (15.1%) | 0.31 |
| Nasogastric feeding given, n(%) | 103/ 767 (13.4%) | 60/ 497 (12.1%) | 43/ 270 (15.9%) | 0.13 |
| Patient mobilised by therapist, n(%) | 619/ 767 (80.7%) | 397/ 497 (79.9%) | 222/ 270 (82.2%) | 0.43 |
| Compression stockings used, n(%) | 80/ 767 (10.4%) | 52/ 497 (10.5%) | 28/ 270 (10.4%) | 0.97 |
| Subcutaneous heparin used, n(%) | 70/ 774 (9.0%) | 55/ 502 (11.0%) | 15/ 272 (5.5%) | 0.01 |
| Any antithrombotic agent (antiplatelet or heparin) used in first 24 hours, n(%) | 149/ 774 (19.3%) | 79/ 502 (15.7%) | 70/ 272 (25.7%) | 0.0008 |
| Intravenous steroids administered, n(%) | 11/ 767 (1.4%) | 9/ 497 (1.8%) | 2/ 270 (0.7%) | 0.23 |
| Hemicraniectomy performed, n(%) | 0/0(0.0) | 0/0(0.0) | 0/0(0.0) |  |
| Any neurosurgery performed, n(%) | 41/ 774 (5.3%) | 32/ 502 (6.4%) | 9/ 272 (3.3%) | 0.07 |
| Any stroke unit admission, n(%) | 684/ 767 (89.2%) | 440/ 498 (88.4%) | 244/ 269 (90.7%) | 0.32 |
| Any intensive care unit admission, n(%) | 20/ 766 (2.6%) | 16/ 497 (3.2%) | 4/ 269 (1.5%) | 0.15 |
| Any rehabilitation given, n(%) | 521/ 767 (67.9%) | 310/ 497 (62.4%) | 211/ 270 (78.1%) | <0.0001 |
| Decision to withdrawal active care, n(%) | 21/ 768 (2.7%) | 13/ 498 (2.6%) | 8/ 270 (3.0%) | 0.77 |

Data are n/N (%) or mean (standard deviation).

HSRC: hyperacute stroke research centre; BP: blood pressure.

**Table S2 Key secondary outcome of symptomatic intracerebral haemorrhage across all definitions by hyperacute stroke research centres (HSRCs) and non-HSRCs**

|  | All UK patients  (n=774) | HSRC patients  (n=502) | Non-HSRC patients  (n=272) | P value |
| --- | --- | --- | --- | --- |
| SITS-MOST criteria | 11/ 774 (1.6%) | 8/ 502 (1.6%) | 3/ 272 (1.1%) | 0.58 |
| NINDS criteria | 40/ 774 (5.2%) | 27/ 502 (5.4%) | 13/ 272 (4.8%) | 0.72 |
| ECASS-2 criteria | 25/ 774 (3.2%) | 17/ 502 (3.4%) | 8/ 272 (2.9%) | 0.74 |
| ECASS-3 criteria | 11/ 774 (1.4%) | 7/ 502 (1.4%) | 4/ 272 (1.5%) | 0.93 |
| IST-3 criteria | 16/ 774 (2.1%) | 10/ 502 (2.0%) | 6/ 272 (2.2%) | 0.84 |
| Clinician-reported | 60/ 774 (7.8%) | 40/ 502 (8.0%) | 20/ 272 (7.4%) | 0.76 |
| Fatal ICH | 6/ 774 (0.8%) | 4/ 502 (0.8%) | 2/ 272 (0.7%) | 0.93 |
| Adjudicated any ICH | 117/ 774 (15.1%) | 73/ 502 (14.5%) | 44/ 272 (16.2% | 0.54 |

Data are n/ N (%).

HSRC: hyperacute stroke research centres; SITS-MOST: Safe Implementation of Thrombolysis in Stroke Monitoring Study; NINDS: National Institute of Neurological Disorders and Stroke; ECASS: European Co-operative Acute Stroke Study; IST: International Stroke Trial; ICH: intracerebral haemorrhage.

**Table S3 Key efficacy outcomes by randomised treatment and hyperacute stroke research centres (HSRCs) and non-HSRCs**

|  | Randomised treatment, n(%) | | OR | P trend | AOR^1^ | P trend | AOR^2^ | P trend |
| --- | --- | --- | --- | --- | --- | --- | --- | --- |
|  | Low-dose | Standard-dose |  |  |  |  |  |  |
| Death or disability (mRS score 2+3+4+5+6) | | | | | | | | |
| Non-HSRC sites | 85/128(66.4%) | 80/ 128(62.5%) | 0.84  (0.51 -1.41) | 0.81 | 0.79  (0.44 -1.43) | 0.68 | 0.76  (0.39 -1.49) | 0.54 |
| HSRC sites | 132/234(56.4%) | 117/233(50.2%) | 0.78  (0.54 -1.12) |  | 0.69  (0.46 -1.04) |  | 0.59  (0.36 -0.94) |  |
|  |  |  |  |  |  |  |  |  |
| Death or major disability (mRS score 3+4+5+6) | | | | | | | | |
| Non-HSRC sites | 60/128(46.9%) | 61/128(47.7%) | 1.03  (0.63 -1.69) | 0.51 | 1  (0.55 -1.82) | 0.33 | 0.78  (0.39 -1.54) | 0.55 |
| HSRC sites | 94/234(40.2%) | 84/233(36.1%) | 0.84  (0.58 -1.22) |  | 0.72  (0.46 -1.11) |  | 0.62  (0.37 -1.05) |  |
|  |  |  |  |  |  |  |  |  |
| Death at day 90(mRS score 6) | | | | | | | | |
| Non-HSRC sites | 13/136(9.6%) | 21/136(15.4%) | 1.73  (0.83 -3.61) | 0.81 | 2.05  (0.86 -4.87) | 0.51 | 2.56  (0.84 -7.81) | 0.66 |
| HSRC sites | 20/253(7.9%) | 29/249(11.6%) | 1.54  (0.84 -2.79) |  | 1.4  (0.71 -2.77) |  | 1.39  (0.51 -3.77) |  |

HSRC: hyperacute stroke research centre; mRS: modified Rankin scale.

Model 1: adjusted analysis for minimisation variables including NIHSS score and time from stroke onset to randomisation, and baseline variables: age, sex, ethnicity, systolic blood pressure, heart rate, hypercholesterolaemia, current smoker, premorbid mRS, premorbid use of antihypertensive therapy, aspirin or other antiplatelet agent, and randomised treatment (low-dose versus standard-dose).

Model 2: as Model 1, plus systolic blood pressure at 24 hours, fever occurrence, nasogastric feeding given, subcutaneous heparin used, patient mobilized by therapist, any stroke unit admission, any neurosurgery performed, and any rehabilitation given.

**Table S4 Key efficacy outcomes by randomised treatment and hyperacute stroke research centres (HSRCs) and non-HSRCs**

|  | Randomised treatment | mRS categories (unadjusted) | | | | | | | OR | P interaction | OR | P interaction | OR | P interaction |
| --- | --- | --- | --- | --- | --- | --- | --- | --- | --- | --- | --- | --- | --- | --- |
|  |  | 0 | 1 | 2 | 3 | 4 | 5 | 6 |  |  |  |  |  |  |
| Non-HSRC sites | Low-dose (n=128) | 20  (15.6%) | 23  (18.0%) | 25  (19.5%) | 25  (19.5%) | 13  (10.2%) | 9  (7.0%) | 13  (10.2%) | 1.11  (0.72 -1.7) | 0.41 | 1.05  (0.67 -1.65) | 0.58 | 0.89  (0.55 -1.44) | 0.81 |
|  | Standard-dose (n=128) | 17  (13.3) | 31  (24.2%) | 19  (14.8%) | 20  (15.6%) | 10  (7.8%) | 10  (7.8%) | 21  (16.4%) |  |  |  |  |  |  |
| HSRC sites | Low-dose (n=234) | 46  (19.7%) | 56  (23.9%) | 38  (16.2%) | 37  (15.8%) | 25  (10.7%) | 12  (5.1%) | 20  (8.5%) | 0.89  (0.65-1.23) |  | 0.93  (0.67 -1.29) |  | 0.83  (0.58 -1.18) |  |
|  | Standard-dose (n=233) | 54  (23.2%) | 62  (26.6%) | 33  (14.2%) | 24  (10.3%) | 17  (7.3%) | 14  (6.0%) | 29  (12.4%) |  |  |  |  |  |  |
|  |  |  |  |  |  |  |  |  |  |  |  |  |  |  |

HSRC: hyperacute stroke research centre; mRS: modified Rankin scale.

Model 1: adjusted analysis for minimisation variables including NIHSS score and time from stroke onset to randomisation, and baseline variables: age, sex, ethnicity, systolic blood pressure, heart rate, hypercholesterolaemia, current smoker, premorbid mRS, premorbid use of antihypertensive therapy, aspirin or other antiplatelet agent, and randomised treatment (low-dose versus standard-dose).

Model 2: as Model 1, plus systolic blood pressure at 24 hours, fever occurrence, nasogastric feeding given, subcutaneous heparin used, patient mobilised by therapist, any stroke unit admission, any neurosurgery performed, and any rehabilitation given.

**Table S5 Key safety outcome of symptomatic intracerebral haemorrhage by randomised treatment and hyperacute stroke research centres (HSRCs) and non-HSRCs**

|  | Total  (n=774) | HSRC sites  (n=502) | Non-HSRC sites  (n=272) | P value |
| --- | --- | --- | --- | --- |
| SITS-MOST criteria |  |  |  |  |
| Low-dose | 4/ 389 (1.0%) | 3/ 253 (1.2%) | 1/ 136 (0.7%) | 0.67 |
| Standard-dose | 7/ 385 (1.8%) | 5/ 249 (2.0%) | 2/ 136 (1.5%) | 0.71 |
| NINDS criteria |  |  |  |  |
| Low-dose | 19/ 389 (4.9%) | 11/ 253 (4.3%) | 8/ 136 (5.9%) | 0.50 |
| Standard-dose | 21/ 385 (5.5%) | 16/ 249 (6.4%) | 5/ 136 (3.7%) | 0.26 |
| ECASS2 criteria |  |  |  |  |
| Low-dose | 9/ 389 (2.3%) | 6/ 253 (2.4%) | 3/ 136 (2.2%) | 0.92 |
| Standard-dose | 16/ 385 (4.2%) | 11/ 249 (4.4%) | 5/ 136 (3.7%) | 0.73 |
| ECASS3 criteria |  |  |  |  |
| Low-dose | 5/ 389 (1.3%) | 3/ 253 (1.2%) | 2/ 136 (1.5%) | 0.81 |
| Standard-dose | 6/ 385 (1.6%) | 4/ 249 (1.6%) | 2/ 136 (1.5%) | 0.92 |
| IST-3 criteria |  |  |  |  |
| Low-dose | 8/ 389 (2.1%) | 4/ 253 (1.6%) | 4/ 136 (2.9%) | 0.37 |
| Standard-dose | 8/ 385 (2.1%) | 6/ 249 (2.4%) | 2/ 136 (1.5%) | 0.54 |
| Clinician-reported |  |  |  |  |
| Low-dose | 28/ 389 (7.2%) | 17/ 253 (6.7%) | 11/ 136 (8.1%) | 0.62 |
| Standard-dose | 32/ 385 (8.3%) | 23/ 249 (9.2%) | 9/ 136 (6.6%) | 0.37 |
| Fatal ICH |  |  |  |  |
| Low-dose | 2/ 389 (0.5%) | 2/ 253 (0.8%) | 0/ 0 (0%) | 0.30 |
| Standard-dose | 4/ 385 (1.0%) | 2/ 249 (0.8%) | 2/ 136 (1.5%) | 0.54 |
| Adjudicated any ICH |  |  |  |  |
| Low-dose | 51/ 389 (13.1%) | 29/ 253 (11.5%) | 22/ 136(16.2%) | 0.19 |
| Standard-dose | 66/ 385 (17.1%) | 44/ 249 (17.7%) | 22/ 136(16.2%) | 0.71 |
| Any ICH |  |  |  |  |
| Low-dose | 60/ 389 (15.4%) | 36/ 253 (14.2%) | 24/ 136(17.6%) | 0.37 |
| Standard-dose | 72/ 385 (18.7%) | 48/ 249 (19.3%) | 24/ 136(17.6%) | 0.70 |

Data are n/ N (%).

HSRC: hyperacute stroke research centres; SITS-MOST: Safe Implementation of Thrombolysis in Stroke Monitoring Study; NINDS: National Institute of Neurological Disorders and Stroke; ECASS: European Co-operative Acute Stroke Study; IST: International Stroke Trial; ICH: intracerebral haemorrhage.
